# Supplementary material for: Age-Related Influence on Static and Dynamic Balance Abilities: An Inertial Measurement Unit-Based Evaluation
Source: Sensors (Basel). 2024 Nov 3;24(21):7078. doi: 10.3390/s24217078 (PMC11548656; doi:10.3390/s24217078)
Supplement: Supplementary file 1 [file sensors-24-07078-s001.zip › sensors-3199742-supplementary.pdf]

Table S1. Analysis of balance-board stance test with feet-apart stance.

$J_{balance}$	Young	Middle-aged	Older	*p*-value	Post-hoc analysis	
Waist	1.271 (1.022)	1.371 (1.057)	1.896 (1.247)	0.1184	Y-M	0.8212
					Y-O	0.0736
					M-O	0.0878

Data are median (IQR). Y-M: Young vs Middle-aged; Y-O: Young vs Older; M-O: Middle-aged vs Older. Bonferroni correction was applied for multiple comparisons, adjusting the *p*-value threshold for significance to 0.05/3 = 0.0167.

Table S2. Analysis of balance-board stance test with feet-together stance.

$J_{balance}$	Young	Middle-aged	Older	*p*-value	Post-hoc analysis	
Waist	1.215 (1.301)	1.514 (1.312)	1.567 (1.077)	0.4800	Y-M	0.6244
					Y-O	0.2352
					M-O	0.4467

Data are median (IQR). Y-M: Young vs Middle-aged; Y-O: Young vs Older; M-O: Middle-aged vs Older. Bonferroni correction was applied for multiple comparisons, adjusting the *p*-value threshold for significance to 0.05/3 = 0.0167.

Table S3. Analysis of single-leg stance test with right foot stance.

$J_{balance}$	Young	Middle-aged	Older	*p*-value	Post-hoc analysis	
Waist	1.026 (0.863)	1.399 (1.141)	1.723 (1.258)	0.3995	Y-M	0.6415
					Y-O	0.1926
					M-O	0.3603

Data are median (IQR). Y-M: Young vs Middle-aged; Y-O: Young vs Older; M-O: Middle-aged vs Older. Bonferroni correction was applied for multiple comparisons, adjusting the *p*-value threshold for significance to 0.05/3 = 0.0167.

Table S4. Analysis of single-leg stance test with left foot stance.

$J_{balance}$	Young	Middle-aged	Older	*p*-value	Post-hoc analysis	
Waist	1.376 (0.735)	1.180 (1.484)	1.973 (1.484)	0.0685	Y-M	0.9097
					Y-O	0.0711
					M-O	0.0353

Data are median (IQR). Y-M: Young vs Middle-aged; Y-O: Young vs Older; M-O: Middle-aged vs Older. Bonferroni correction was applied for multiple comparisons, adjusting the *p*-value threshold for significance to 0.05/3 = 0.0167.
